# Clinical study on the necessity and feasibility of routine MRCP in patients with cholecystolithiasis before LC

**DOI:** 10.1186/s12876-023-03117-3

**Published:** 2024-01-09

**Authors:** Xu Guo, Qing Fan, Yiman Guo, Xinming Li, Jili Hu, Zhuoyin Wang, Jing Wang, Kai Li, Nengwei Zhang, Buhe Amin, Bin Zhu

**Affiliations:** 1 Department of General Surgery, Beijing Shijitan Hospital, Capital Medical University, Tieyi Road 10th, Yangfangdian Street, Haidian District, 100038 Beijing, China; 2 School of Clinical Medicine, Hebei University, Wusi East Road 180th, Lianchi District, Hebei Province 071000 Baoding City, China; 3 Department of Urology, Fuyang People’s Hospital, Anhui Medical University, Sanqing Road 501th, Ying Zhou District, 236012 Fuyang City, Anhui Province China; 4 Department of Hepatobiliary Surgery, The First Affiliated Hospital of Zhengzhou University, No.1 Jianshe Dong Road, ErQi District, 450052 Zhengzhou City, Henan Province China

**Keywords:** Cholecystolithiasis, Magnetic resonance cholangiopancreatography (MRCP), Laparoscopic cholecystectomy (LC), Preoperative routine MRCP

## Abstract

**Background:**

In the past quite a long time, intraoperative cholangiography(IOC)was necessary during laparoscopic cholecystectomy (LC). Now magnetic resonance cholangiopancreatography (MRCP) is the main method for diagnosing common bile duct stones (CBDS). Whether MRCP can replace IOC as routine examination before LC is still inconclusive. The aim of this study was to analyze the clinical data of patients undergoing LC for cholecystolithiasis, and to explore the necessity and feasibility of preoperative routine MRCP in patients with cholecystolithiasis.

**Methods:**

According to whether MRCP was performed before operation, 184 patients undergoing LC for cholecystolithiasis in the Department of General Surgery, Beijing Shijitan Hospital, Capital Medical University from January 1, 2017 to December 31, 2018 were divided into non-MRCP group and MRCP group for this retrospective study. The results of preoperative laboratory test, abdominal ultrasound and MRCP, biliary related comorbidities, surgical complications, hospital stay and hospitalization expenses were compared between the two groups.

**Results:**

Among the 184 patients, there were 83 patients in non-MRCP group and 101 patients in MRCP group. In MRCP group, the detection rates of cholecystolithiasis combined with CBDS and common bile duct dilatation by MRCP were higher than those by abdominal ultrasound (*P* < 0.05). The incidence of postoperative complications in non-MRCP group (8.43%) was significantly higher (*P* < 0.05) than that in MRCP group (0%). There was no significant difference in hospital stay (*P* > 0.05), but there was significant difference in hospitalization expenses (*P* < 0.05) between the two groups. According to the stratification of gallbladder stone patients with CBDS, hospital stay and hospitalization expenses were compared, and there was no significant difference between the two groups (*P* > 0.05).

**Conclusions:**

The preoperative MRCP can detect CBDS, cystic duct stones and anatomical variants of biliary tract that cannot be diagnosed by abdominal ultrasound, which is helpful to plan the surgical methods and reduce the surgical complications. From the perspective of health economics, routine MRCP in patients with cholecystolithiasis before LC does not increase hospitalization costs, and is necessary and feasible.

## Introduction

Laparoscopic cholecystectomy (LC) has become the standard treatment for patients with gallstones [1]. For most patients with cholecystolithiasis, preoperative abdominal ultrasound can confirm the diagnosis [2, 3]. But the results of ultrasonography are easily influenced by the gastrointestinal gas of patients and the skill level of operators, and it is easy to miss combined common bile duct stones (CBDS) and difficult to detect the anatomical variation of bile duct. In the past quite a long time, intraoperative cholangiography(IOC)was necessary during LC. At present, magnetic resonance cholangiopancreatography (MRCP) is the main method for diagnosing CBDS [4, 5]. MRCP is typically only necessary when abdominal ultrasound reveals a dilated common bile duct exceeding 6 mm in diameter, the patient exhibits jaundice and elevated transaminase levels, or a history of pancreatitis exists [6]. MRCP before LC has the following advantages in theory. First, MRCP can diagnose CBDS and common bile duct dilatation that cannot be detected by abdominal ultrasound, and clarify the cause of biliary obstruction [7]. Second, MRCP can fully understand the anatomical variation of extrahepatic bile duct and the severity of gallbladder inflammation, which can assess the difficulty of surgery and reduce conversion to laparotomy and complications such as bile duct injury [8]. Third, MRCP is helpful for the diagnosis of gallbladder cancer and other biliary and pancreatic diseases [9]. However, there is no strong evidence to support or refute whether MRCP before LC is suitable for all patients with cholecystolithiasis and the cost of MRCP match its benefits (price-performance ratio) at present. In this study, we analyzed the clinical data of 184 patients with cholecystolithiasis treated by LC and discussed the necessity and feasibility of preoperative routine MRCP by a “real world” study.

## Patients and methods

We conducted a retrospective cohort study to analyze the clinical data of patients who underwent LC for cholecystolithiasis in the Department of General Surgery, Beijing Shijitan Hospital, Capital Medical University from January 1, 2017 to December 31, 2018. According to whether MRCP was performed before operation, patients were divided into non-MRCP group and MRCP group for this study. Only abdominal ultrasonography was performed in non MRCP group, and both abdominal ultrasonography and MRCP were performed in MRCP group. The selected patients for enrollment were adults (age 18 and older), who were diagnosed as gallstones by abdominal ultrasound before operation, and received LC or LC combined with other stone extraction. But patients with the following conditions need to be excluded. First, patient underwent LC for gallbladder cancer. Secondary, patients had cardiopulmonary and other organ insufficiency. Third, patients had some items missing from the case data. This study finally included 184 cases.

The relevant research content data were collected mainly through the hospital medical record center and the medical record query system, and the clinical data of the two groups were compared to explore the necessity and feasibility of preoperative routine MRCP in patients with cholecystolithiasis. The main evaluating indicators were the results of abdominal ultrasound and MRCP, incidence of biliary related comorbidities and postoperative complications of LC. The secondary evaluating indicators were including the hospitalization-related information (hospital stay, hospitalization costs) and the preoperative relevant laboratory tests, such as white blood cell count (WBC), neutrophil percentage (N%), alanine aminotransferase (ALT), aspartate aminotransferase (AST), alkaline phosphatase (ALP), gamma-glutamyl transpeptidase (γ-GT), direct bilirubin (D-Bil), indirect bilirubin (I-Bil) and total bilirubin (T-Bil).

Statistical analysis was performed by using the SPSS software (version 26.0, Armonk, NY: IBM Corp). Continuous variable data were compared by the t-test. Categorical variable data were compared by the χ2 test. If any cell had expected count less than 5, Fisher’s test was analyzed. *P*<0.05 was considered statistically significant difference.

## Results

### Basic information and preoperative laboratory examination

Among the 184 patients, there were 83 patients in non-MRCP group and 101 patients in MRCP group. There was no significant difference (*P* > 0.05) in general demographic data such as age, gender and body mass index (BMI) between the two groups (Table 1).

**Table 1:** Comparison of basic information and preoperative laboratory examination data between the two groups

	Non-MRCP group(n = 83)	MRCP group (n = 101)	T/Z	*P*
Age (years)	57.33 ± 14.72(mean ± SD) 22–86	60.03 ± 15.61 23–89	-1.200	0.232
Gender				
Male	30	48	2.416	0.120
Female	53	53		
Height (cm)	165.20 ± 6.79	166.72 ± 7.61	-1.412	0.160
Body weight (kg)	67.44 ± 11.63	69.40 ± 11.81	-1.132	0.259
BMI(kg/m^2^)	24.67 ± 3.81	24.88 ± 3.28	-0.392	0.695
T(℃)	36.44 ± 0.45	36.42 ± 0.34	0.401	0.689
WBC(*10^9^)	5.66 ± 1.53	5.78 ± 1.72	-0.509	0.612
N%	58.59 ± 10.36	58.90 ± 11.58	-0.192	0.848
ALT(u/L)	28.48 ± 20.86	34.97 ± 31.18	-1.621	0.107
AST(u/L)	26.34 ± 17.63	26.76 ± 20.56	-0.149	0.882
ALP(u/L)	70.25 ± 23.69	76.06 ± 26.29	-1.558	0.121
γ-GT(u/L)	42.83 ± 33.29	46.07 ± 35.30	-0.635	0.526
D-Bil(umol/l)	5.73 ± 3.99	7.11 ± 5.34	-1.956	0.052
I-Bil(umol/l)	8.14 ± 3.53	9.69 ± 4.65	-2.497	0.013
T-Bil(umol/l)	13.87 ± 5.44	16.80 ± 8.14	-2.805	0.006

Note: Normal values for each indicator: T (axillary temperature)37 <°C; WBC 4–10*10^9^/L; N% 50-70%; ALT 7–40 u/L; AST 13–35 u/L; γ-GT 0–50 u/L; ALP 40–110 u/L; D-Bil 0–8 umol/l (or 0-0.47 mg/dL); I-Bil 1–14 umol /l (or 0-0.82 mg/dL); T-Bil 0–23 umol/l (or 0-1.35 mg/dL)

There was no significant difference in preoperative axillary temperature (T), liver function and D-Bil results between the two groups (*P* > 0.05). While there was a statistically significant difference in I-Bil and T-Bil between the two groups (*P* < 0.05), the two indicators were higher in the MRCP group than in the non-MRCP group (Table 1).

### Clinical diagnosis, imaging examination and surgical-related information

The biliary related comorbidities and ultrasonography results of the 83 patients with cholecystolithiasis in non-MRCP group were shown in Table 2. Among them, 76 patients underwent simple LC surgery. Six cases of CBDS with common bile duct dilatation (one of them combined with preoperative percutaneous gallbladder puncture and drainage) underwent LC combined with common bile duct exploration, choledocholithotomy and T-tube drainage. One case of CBDS without common bile duct dilatation underwent LC combined with preoperative Endoscopic retrograde cholangiopancreatography (ERCP), Endoscopic sphincteropapillotomy (EST) lithotomy.

**Table 2:** Comparison of clinical diagnosis, imaging examination and surgery-related information between the two groups

	Non-MRCP group(n = 83)	MRCP group (n = 101)	χ2/Z	*P*
Biliary related comorbidities				
Cholecystitis	73(87.95%)	94(93.07%)	1.423	0.233
CBDS	7(8.43%)	21(20.79%)	5.393	0.020
Cholangitis	3(3.61%)	6(5.94%)	-0.726	0.468
Gallbladder duct stones	2(2.40%)	6(5.94%)	-1.166	0.244
Adenomyomatosis of gallbladder	2(2.40%)	10(9.90%)	-2.042	0.041
Biliary pancreatitis	0	3(2.97%)	-1.579	0.114
Gallbladder polyps	4(4.82%)	5(4.95%)	-0.041	0.967
Mirizzi syndrome	1(1.20%)	3(2.97%)	-0.815	0.415
Abdominal ultrasound findings	83	101		
CBDS	7(8.43%)	10(9.90%)	0.117	0.732
Dilation of the common bile duct	6(7.23%)	10(9.90%)	0.410	0.522
Biliary tract variation	0	0		
Biliary tract tumors	0	0		
LC combined surgery				
Common bile duct exploration	6(7.23%)	15(14.85%)	2.618	0.106
Common bile duct incision	6(7.23%)	15(14.85%)	2.618	0.106
T-tube drainage	6(7.23%)	15(14.85%)	2.618	0.106
Preoperative cholecystocentesis drainage	1*(1.20%)	1*(0.99%)	-0.139	0.889
Preoperative ERCP + EST stone extraction	1(1.20%)	7*(6.93%)	-1.890	0.059
LC intraoperative situation				
CBDS	6(7.23%)	15(14.85%)	2.618	0.106
Dilation of the common bile duct	6(7.23%)	15(14.85%)	2.618	0.106
Biliary tract variation	1(1.20%)	5(4.95%)	-2.050	0.040
Biliary tract tumors	0	0		
Operating time (min)	78.48 ± 49.54	89.26 ± 48.31	-1.488	0.138

Note: Dilated common bile duct: diameter of common bile duct > 6 mm. *One patient in non-MRCP group and one patient in MRCP group underwent LC combined with preoperative percutaneous cholecystocentesis and drainage, common bile duct exploration, choledocholithotomy and T-tube drainage; one patient in the MRCP group underwent LC combined with preoperative ERCP + EST lithotomy, common bile duct exploration, choledochotomy and T-tube drainage

The biliary related comorbidities, ultrasound and MRCP results of 101 patients with cholecystolithiasis in MRCP group were shown in Tables 2 and 3. Of the 101 cases, 80 cases underwent simple LC surgery. Fifteen cases of CBDS with common bile duct dilatation (one of them combined with preoperative percutaneous gallbladder puncture and drainage) underwent LC combined with common bile duct exploration, choledocholithotomy and T-tube drainage. Seven cases without common bile duct dilatation (one case combined with common bile duct exploration, common bile duct lithotomy and T-tube drainage) underwent LC combined with preoperative ERCP, EST stone extraction.

**Table 3:** Comparison of abdominal ultrasound and MRCP findings in the MRCP group

Inspection findings	ultrasonography	MRCP
CBDS	10/101(9.90%)	21/101(20.79%)
Gallbladder duct stones	0	6/101(5.94%)
Adenomyomatosis of gallbladder	0	10/101(9.90%)
Biliary pancreatitis	0	3/101(2.97%)
Mirizzi syndrome	0	3/101(2.97%)
Biliary tract tumors	0	0
Dilation of the common bile duct	10/101(9.90%)	15/101(14.85%)
Biliary tract variation	0	13/101(12.87%)

There was a statistically significant difference in CBDS, adenomyomatosis and biliary tract variation between the two groups (*P* < 0.05). MRCP can find more CBDS, adenomyomatosis and biliary tract variation than ultrasound.

In MRCP group, the detection rate of preoperative MRCP in the diagnosis of cholecystolithiasis with CBDS, cystic duct stones, gallbladder adenomyosis, common bile duct dilatation and biliary tract variation was higher than those of abdominal ultrasound. There was significant difference (*P* < 0.05) in the detection rate of CBDS and common bile duct dilatation between MRCP and abdominal ultrasound (Tables 3, 4 and 5).

**Table 4:** Diagnosis of CBDS by abdominal ultrasound with MRCP in the MRCP group

ultrasonography	MRCP		Total	χ2/Z	*P*
	+	-			
+	10	0	10	-3.317	0.001
-	11	80	91		
Total	21	80	101		

**Table 5:** Diagnosis of common bile duct dilatation by abdominal ultrasound and MRCP in the MRCP group

ultrasonography	MRCP		Total	χ2/Z	*P*
	+	-			
+	10	0	10	-2.236	0.025
-	5	86	91		
Total	15	86	101		

### Postoperative complications

There were no postoperative complications in MRCP group, while there were 7 cases of postoperative complications in non-MRCP group. Among 5 cases with comorbidities of residual CBDS, one patient developed obstructive jaundice after LC, and then ERCP + EST stone removal was performed, who was hospitalized for 18 days at a total cost of 44,277.49 yuan (RMB). One patient was readmitted due to acute pancreatitis after LC, and then underwent ERCP + EST stone extraction and was hospitalized again for 16 days at a cost of 20,878.72 yuan. Two patients were readmitted due to biliary colic after LC, and underwent ERCP + EST stone extraction for 8 days and 9 days at a cost of 12,389.42 yuan and 13,090.56 yuan respectively. One patient had biliary colic symptoms after LC. MRCP found that the residual CBDS was very small, and there was no common bile duct dilatation. After a period of observation, MRCP was performed again, indicating no stones. In addition, one patient with biliary colic after LC was found to be cystic duct residual gallstones by MRCP, who was hospitalized for surgery again and recovered after stone removal. The other patient with biliary tract variation had intraoperative biliary tract injury, and no biliary tract variation was found by preoperative ultrasonography. After Rou-x surgery, he recovered and was hospitalized for 20 days at a cost of 24,366.67 yuan.

Comparing the postoperative complications between the two groups, the non-MRCP group (7/83, 8.43%) was significantly higher than the MRCP group (0/101) with a statistically significant difference (*P* < 0.05) in Table 6.

**Table 6:** Comparison of postoperative complications between the two groups

Complications	Group		Total	χ2/Z	*P*
	Non-MRCP group	MRCP group			
Yes	7	0	7	-2.968	0.003
No	76	101	177		
Total	83	101	184		

### Hospital stay and hospitalization expenses

There was no significant difference in hospital stay (*P* > 0.05), but there was a statistically significant difference in hospitalization costs (*P* < 0.05) between the two groups. According to whether patients with CBDS, the hospital stay and hospitalization costs were stratified and analyzed. In non-MRCP group, there were 7 patients with CBDS and 76 patients without CBDS. In MRCP group, there were 21 patients with CBDS and 80 patients without CBDS. There was no significant difference (*P* > 0.05) in hospital stay and costs between the two groups (Table 7).

**Table 7:** Comparison of hospital stay and hospitalization expenses between the two groups

	Non-MRCP group(n = 83)	MRCP group (n = 101)	*t*	*P*
Hospital stay (day)	8.28 ± 4.10	9.18 ± 4.73	-1.365	0.174
Hospitalization costs (ten thousand yuan)	1.67 ± 0.60	1.92 ± 0.65	-2.625	0.009
Hospital stay of patients with CBDS (day)	14.57 ± 2.76	14.85 ± 5.04	-0.142	0.888
Hospitalization costs of patients with CBDS (Ten thousand yuan)	2.50 ± 0.53	2.74 ± 0.53	-1.050	0.303
Hospital stay of patients without CBDS (day)	7.70 ± 3.71	7.69 ± 3.32	0.018	0.986
Hospitalization costs of patients without CBDS (Ten thousand yuan)	1.60 ± 0.55	1.70 ± 0.48	-1.255	0.211

## Discussion

LC has become the first choice for the treatment of cholecystolithiasis [10–13]. However, the postoperative residual CBDS and bile duct injury after LC cannot be ignored [14]. It is reported that 10% ~ 20% of patients with cholecystolithiasis coexist with CBDS [15]. If the presence of CBDS can be determined before LC, the incidence of postoperative residual CBDS can be reduced [16]. The incidence of LC bile duct injury is 0.2–1.1% [17]. Although there is a “learning curve” factor in the cause of bile duct injury, bile duct anatomical factors such as bile duct anatomical variation, acute inflammation of the gallbladder and Mirizzi syndrome are also factors that cannot be ignored for bile duct injury, which increase the difficulty of surgery [18, 19]. Adequate preoperative knowledge of the bile duct-related anatomy and prediction of LC difficulty can help to reduce the occurrence of complications.

At present, preoperative prediction of the possibility of LC in patients with cholecystolithiasis mainly relies on the results of medical history, laboratory examinations and imaging examinations. Abdominal ultrasound is helpful for the accurate diagnosis of cholecystolithiasis and cholecystitis, and the diagnostic accuracy of common bile duct dilatation can also reach 77–87% [20]. The accuracy of abdominal CT in the diagnosis of cholecystolithiasis and CBDS is higher than that of abdominal ultrasound. Abdominal CT is not the first choice for diagnosing cholecystolithiasis because it is difficult to diagnose stones with low calcium content and due to the influence of radiation and other factors [4]. IOC has always been an effective method for the diagnosis of CBDS [21]. However, IOC lengthens LC, complicates the surgery and possibly introduces more opportunities for detrimental outcomes. Preoperative MRCP has a tendency to replace IOC in recent years.

ASGE guidelines predict the probability of CBDS according to different clinical indicators, which are divided into three levels. First, very strong predictive factors for choledocholithiasis include evidence of CBDS in abdominal ultrasound. Second, strong predictive factors for choledocholithiasis include common bile duct diameter > 6 mm (with gallbladder in situ), total serum bilirubin > 4 mg/dL or bilirubin level 1.8 to 4 mg/dL. Third, moderate predictive factors for choledocholithiasis include abnormal liver biochemical test other than bilirubin, age older than 55 years or clinical gallstone pancreatitis [21–23]. According to the above prediction indicators, the ASGE guidelines also classify the classes for CBDS and give corresponding treatment suggestions. Patients with very strong predictive factors for choledocholithiasis belong to the high risk classes (> 50%) for choledocholithiasis and need to be clearly diagnosed by ERCP, and treated accordingly. Patients with predictors strong and moderate predictive factors for choledocholithiasis belong to the intermediate risk classes (10%~50%) for choledocholithiasis and should undergo preoperative MRCP, endoscopic ultrasound (EUS), IOC, or laparoscopic ultrasound depending on the local expertise and availability. Patients with no predictive factors for choledocholithiasis belong to the low risk classes (<10%) for choledocholithiasis and can choose to undergo LC directly [23, 24]. The clinical guideline recommends that patients with gallstone at moderate risk or above of CBDS undergo further preoperative examinations such as MRCP before LC. Should patients with low risk for CBDS (< 10%) and no predictors undergo preoperative MRCP in addition to abdominal ultrasound? This is the problem to be solved in this study.

An important reason for residual CBDS after LC is insufficient preoperative examination. Abdominal ultrasound has high specificity in the diagnosis of CBDS, but the sensitivity is only 22%~55%. It is more difficult to find distal CBDS without bile duct dilatation and anatomical variation of bile duct. Studies showed that the sensitivity and specificity of MRCP examination for the diagnosis of gallbladder and bile duct diseases could reach 90–100% [24]. MRCP can clearly display the image of the “biliary tree” and obtain the effect similar to direct cholangiography that the surgeon wants to know. At the same time, MRCP can accurately show the image information such as the size, number, location of the bile duct stones and the degree of biliary tract dilatation [25, 26]. For solitary stones or CBDS smaller than 6 mm detected by MRCP, they can spontaneously pass from the common bile duct into the duodenum. Recognizing this possibility, Saito et al. advocated for regular MRCP follow-up instead of unnecessary invasive procedures such as ERCP [27]. Many patients in our study benefited from MRCP examination. In MRCP group, there were 21 patients combined with CBDS. Preoperative abdominal ultrasound revealed only 10 of these patients with CBDS, all of whom were dilatation of common bile duct; while MRCP diagnosed all cases of CBDS, including 15 cases of common bile duct dilatation, 6 cases without common bile duct dilatation, jaundice and abnormal liver function. MRCP showed CBDS and bile duct dilatation that were not diagnosed by abdominal ultrasound, especially in cases of CBDS with normal liver function, no jaundice and no bile duct dilatation. MRCP can make up for the defect that abdominal ultrasound cannot show the deep extrahepatic bile duct.

Residual CBDS after LC also has the risk of causing other gallstone-related complications, such as early bile leakage, obstructive jaundice, cholangitis and pancreatitis, which results in prolonged hospitalization or readmission and consequently increase medical costs. In this study, one patient in non-MRCP group developed obstructive jaundice after LC, who was diagnosed with residual CBDS and cured by a further EST stone extraction. Another patient developed acute pancreatitis due to residual CBDS after LC and was readmitted to the hospital, who also recovered after EST stone extraction.

Postoperative complications such as residual cystic duct stones or residual small gallbladder often occur when the cystic duct is tortuously too long or too short. In addition, the complications also occur in patients with low openings and openings behind or on the left side of the common bile duct [28]. Preoperative MRCP can help the surgeon understand the relationship between the cystic duct, the common hepatic duct and the common bile duct, the length of the cystic duct and the presence of stones. In this study, preoperative MRCP revealed cystic duct stones in 6 cases, which were treated in a targeted manner during surgery to avoid the occurrence of residual stones in the cystic duct. In non-MRCP group, the residual cystic duct stones were found by postoperative MRCP in one case of right upper abdominal pain after LC and the symptoms were relieved after the second operation.

Anatomical variation of the bile duct, acute inflammation of the gallbladder, stone incarceration in the gallbladder neck, gallbladder-colic fistula and Mirizzi syndrome will increase the difficulty of surgery, which are also factors that can easily cause bile duct injury in LC [18, 19, 28]. The surgeon can objectively judge the severity of cholecystitis and the thickness of the gallbladder wall by MRCP, which is helpful to estimate the difficulty of surgery, prevent bile duct injury and reduce the incidence of conversion to laparotomy [7]. Preoperative MRCP is also helpful for the diagnosis of Mirizzi syndrome and the selection of appropriate surgical methods to minimize the occurrence of bile duct injury [29]. In non MRCP group, one patient with common bile duct injury failed to detect the bile duct variation by preoperative abdominal ultrasound, which may be one of the reasons for bile duct injury. In MRCP group, preoperative MRCP revealed there were pancreaticobiliary variants in 13 patients, including the variation of cystic duct passed through the back of common bile duct or flowed into common hepatic duct, accessory pancreatic duct and intrahepatic bile duct. The surgeon fully understood the anatomical structure of biliary system before operation, so as to effectively prevent the occurrence of complications such as intraoperative biliary injury during the operation. Three patients with Mirizzi syndrome were diagnosed by preoperative MRCP in this study. Because of proper intraoperative management, there were no complications such as biliary tract injury.

In addition, MRCP before LC is also helpful for the diagnosis of pancreatitis and biliopancreatic tumors [30, 31]. In our study, there were 3 patients with biliary colic in the MRCP group. Preoperative MRCP identified acute pancreatitis that was not diagnosed by abdominal ultrasound. The patients received conservative treatment before LC, avoiding LC in the acute stage of pancreatitis.

Therefore, MRCP before LC in patients with cholecystolithiasis can determine whether CBDS is combined, and fully understand the anatomy of the biliary tract and the degree of gallbladder inflammation. It is helpful to predict the difficulty of surgery and reduce the occurrence of complications such as residual CBDS and bile duct injury after LC.

Bile duct injury is a major complication after LC surgery, mainly due to the misidentification of anatomy and variation of the bile duct [32]. MRCP can clearly display the anatomy and variation of the biliary tract in most patients. But in scenarios of complex cases or poor MRCP imaging, multimodal imaging technology such as intraoperative near-infrared fluorescent cholangiography (NIRF-C) combined with preoperative MRCP are very useful for better definition of biliary anatomy [33, 34]. Research has shown that the use of NIRF-C with indocyanine green intraoperatively markedly enhances biliary-structure visualization, which can significantly reduce the bile duct injury and the proportion of conversion to open surgery [35].

Although routine MRCP before LC has certain clinical value for cholecystolithiasis, it increases the cost of examination. How is it cost-effective? Due to the different degree of illness of the two groups of patients, there were more patients with CBDS in MRCP group, which led to an increase in the hospitalization costs compared with non-MRCP group. But according to whether patients of cholecystolithiasis were combined with CBDS or not, the hospital stay and expenses of the two groups were compared and there was no significant difference, suggesting that MRCP did not significantly increase length of stay and the hospitalization costs. The cost of MRCP examinations is very cheap now. It is worthwhile to perform preoperative MRCP, compared with the possible complications and damage to patients without preoperative MRCP. In addition, medical insurance only covers the cost of hospitalization in China, so patients undergoing surgery have a longer hospital stay. From the perspective of health economics, MRCP has obvious advantages in terms of cost-effectiveness. We believe that routine MRCP before LC for gallstones is necessary and feasible.

However, this study also has certain limitations. This is a retrospective study. For one thing, the preoperative I-Bil and T-Bil of the two groups are statistically significant. For another, the ultrasound and MRCP between the two groups cannot be compared horizontally. The diagnostic effects of the two examinations can be compared only by patients in the MRCP group. In addition, the cost-analysis in this study was limited to China, and the applicability of our results to other countries still need to be discussed. Therefore, multicenter studies from different countries are needed to confirm the feasibility of preoperative MRCP before all cholecystectomies in terms of healthcare costs. There are also certain biases and confounding factors. Whether patients with gallstones should undergo routine MRCP before LC needs to be discussed in a follow-up control study with better settings.

In conclusion, routine MRCP in patients with gallstone before LC is a rational use of medical resources and is helpful to further clarify the diagnosis of diseases such as CBDS and understand the anatomy of biliary tract, which has certain guiding significance for predicting the difficulty of operation. According to the preoperative MRCP results, the surgeon can formulate a reasonable surgical plan to deal with CBDS, cystic duct stones, avoid the injury of bile duct, residual stones, effectively prevent intraoperative and postoperative complications, and reduce the risk of secondary surgery.

## Data Availability

The datasets were available from the corresponding author on reasonable request.
